# Analysis of Indian SARS-CoV-2 Genomes Reveals Prevalence of D614G Mutation in Spike Protein Predicting an Increase in Interaction With TMPRSS2 and Virus Infectivity

**DOI:** 10.3389/fmicb.2020.594928

**Published:** 2020-11-23

**Authors:** Sunil Raghav, Arup Ghosh, Jyotirmayee Turuk, Sugandh Kumar, Atimukta Jha, Swati Madhulika, Manasi Priyadarshini, Viplov K. Biswas, P. Sushree Shyamli, Bharati Singh, Neha Singh, Deepika Singh, Ankita Datey, Kiran Avula, Shuchi Smita, Jyotsnamayee Sabat, Debdutta Bhattacharya, Jaya Singh Kshatri, Dileep Vasudevan, Amol Suryawanshi, Rupesh Dash, Shantibhushan Senapati, Tushar K. Beuria, Rajeeb Swain, Soma Chattopadhyay, Gulam Hussain Syed, Anshuman Dixit, Punit Prasad, Odisha COVID-19 Study Group, Arvind Kumar Singh, ILS COVID-19 Team, Aditi Chatterjee, Sanghamitra Pati, Ajay Parida

**Affiliations:** ^1^Institute of Life Sciences (ILS), Bhubaneswar, India; ^2^Regional Medical Research Centre (RMRC), Bhubaneswar, India

**Keywords:** SARS-CoV-2, COVID-19, phylogeny, India, D614G, viral RNA sequencing, protein-protein interaction

## Abstract

Coronavirus disease 2019 (COVID-19), caused by the severe acute respiratory syndrome coronavirus 2 (SARS-CoV-2) virus, has emerged as a global pandemic worldwide. In this study, we used ARTIC primers–based amplicon sequencing to profile 225 SARS-CoV-2 genomes from India. Phylogenetic analysis of 202 high-quality assemblies identified the presence of all the five reported clades 19A, 19B, 20A, 20B, and 20C in the population. The analyses revealed Europe and Southeast Asia as two major routes for introduction of the disease in India followed by local transmission. Interestingly, the19B clade was found to be more prevalent in our sequenced genomes (17%) compared to other genomes reported so far from India. Haplotype network analysis showed evolution of 19A and 19B clades in parallel from predominantly Gujarat state in India, suggesting it to be one of the major routes of disease transmission in India during the months of March and April, whereas 20B and 20C appeared to evolve from 20A. At the same time, 20A and 20B clades depicted prevalence of four common mutations 241 C > T in 5′ UTR, P4715L, F942F along with D614G in the Spike protein. D614G mutation has been reported to increase virus shedding and infectivity. Our molecular modeling and docking analysis identified that D614G mutation resulted in enhanced affinity of Spike S1–S2 hinge region with TMPRSS2 protease, possibly the reason for increased shedding of S1 domain in G614 as compared to D614. Moreover, we also observed an increased concordance of G614 mutation with the viral load, as evident from decreased Ct value of Spike and the ORF1ab gene.

## Introduction

The coronavirus disease 2019 (COVID-19) pandemic is caused by severe acute respiratory syndrome coronavirus 2 (SARS-CoV-2), a betacoronavirus belonging to the Coronaviridae family. The first occurrence of this novel coronavirus was observed in Wuhan, China, in late December 2019, which later spread globally via human-to-human contact transmission (Lu et al., 2020). According to the World Health Organization (WHO) weekly epidemiological update until September 14, 2020, the virus has spread to more than 180 countries with 28.6 million total confirmed cases and 0.9 million deaths worldwide (World Health Organization, 2020). The first occurrence of a coronavirus case in India was observed in mid-January, but the number of cases started to increase from the first week of March. According to the WHO, the number of cases in India has reached 4.7 million with 78,500 deceased (World Health Organization, 2020).

Genomic studies performed to understand the origin of SARS-CoV-2, a positive single-stranded RNA virus, unraveled that it has a zoonotic origin and is transmitted to humans from bats via Malayan pangolins (Zhang T. et al., 2020). The nucleotide sequence of SARS-CoV-2 is ∼79% similar to SARS-CoV-1 and about 50% with MERS-CoV (Middle East respiratory syndrome coronavirus) (Lu et al., 2020). Approximately 30-kb genome of SARS-CoV-2 features a cap structure in 5′ and 3′ poly (A) like other members of the coronavirus family. A major portion of the genome is covered by two ORFs (ORF1a and ORF1b), which code for 15 non-structural proteins, including crucial proteins required for viral replication, such as viral proteases nsp3, nsp5, and nsp12, also known as RNA-dependent RNA polymerase (RdRP) (Kim et al., 2020). The genomic RNA (gRNA) also codes for structural proteins like Spike protein (S), nucleocapsid protein (N), membrane protein (M), and envelope protein (E), which are required for packaging of the virus and at least accessory proteins, but their ORFs are still not experimentally validated (Kim et al., 2020).

The entry of viral particles in the human body occurs through binding with angiotensin I–converting enzyme 2 (hACE2) receptor present on lung epithelial cells (Hoffmann et al., 2020). The predominant infection site is the respiratory tract due to the route of infection. Other than the lungs, it is known to infect other organs of the body, such as the kidney, liver, and intestine, as ACE2 expression is found to be quite high. After the viral entry into host cells, to initiate viral replication, negative-sense RNA intermediates are first synthesized by RdRP activity; these templates are then utilized for synthesis of gRNA and subgenomic RNAs (Kim et al., 2020). Because of the low fidelity of RdRP, mutations are incorporated with high frequency in gRNA, and such mutations are often known to increase the pathogenicity and fitness of the virus (Graepel et al., 2017; Ferron et al., 2018; Korber et al., 2020).

Recently, a predominant mutation, i.e., D614G in Spike protein, has been identified in virus strains sequenced from European population (Korber et al., 2020). There are several reports depicting that D614G mutation in Spike protein is associated with enhanced infectivity and spread of the virus owing to increased interaction with ACE2 receptor present on host cells (Korber et al., 2020). It has also been indicated that this mutation is present on the S2 domain of the Spike protein that is important for cleavage by TMPRSS2 enzyme for cleavage of S1 to facilitate the fusion of the viral Spike with the host cell membrane (Korber et al., 2020; Zhang L. et al., 2020). It is indeed interesting to understand further at a molecular level the changes in the protein structure induced by this mutation and its functional association with the infectivity and disease severity.

At the same time, it has been reported that D614G mutation co-occurs with three more mutations, i.e., 241 in UTR, 3307, and 14408 (Korber et al., 2020). The functional significance of these co-occurring mutations in evolution and virus selectivity is interesting to understand.

In the present study, we have sequenced 225 COVID-19 isolates from patient samples from the state of Odisha, those migrated from the 13 most affected Indian states as a part of DBT’s PAN-India 1000 SARS-CoV-2 RNA genome sequencing consortium, using an amplicon sequencing–based methodology. The travel history of these patients was collected along with their symptomatic and asymptomatic behavior. We performed phylogenetic analysis from sequenced data to understand the genetic diversity and evolution of SARS-CoV-2 in the Indian subcontinent. From the sequenced isolates, we have identified 247 single-nucleotide variants; most of them are observed in ORF1ab, Spike and nucleocapsid protein coding region. Moreover, we have analyzed the D614G mutations in the samples to obtain information on its evolution in Indian population. Protein-modeling analysis of D614G mutation was carried out to identify the impact on structural changes at protein levels. We further performed protein–protein docking simulation to predict the impact of D614G mutation on the interaction between of wild-type and mutated Spike protein with TMPRSS2 enzyme to assess its impact on binding and perhaps viral infectivity.

## Materials and Methods

### Sample Collection

All hospitalized and quarantined patients (March 2020 to June 2020), based on their clinical symptoms (fever or respiratory symptoms) or travel history, were preliminarily involved in this study. We received throat swabs in viral transport media samples of these patients used for SARS−CoV−2 detection. Patients with missing or with negative SARS−CoV−2 test results were excluded from this study based on Ct values obtained by quantitative polymerase chain reaction (qPCR) of isolated RNA. All patients involved in this study were residents of Odisha, India, during the outbreak period of COVID−19. The samples were collected and processed as per the guidelines of the Institutional Ethics and Biosafety Committee. Institutional Biosafety Committee (IBSC) approval (IBSC file no. V-122-MISC/2007-08/01) was taken before processing the samples in BSL3 laboratory.

### Viral Load Detection

RNA isolation for all the 248 human patients was performed using QIAamp Viral RNA Mini Kit (Qiagen, cat. no. 52906). The isolated RNA was subjected to qPCR for determining viral load by Ct values. For qPCR, we performed one-step multiplex real-time PCR using TaqPath^TM^ 1-Step Multiplex Master Mix (Thermo Fisher Scientific, cat. no. A28526), targeting three different gene-specific primer and probe sets—envelope glycoprotein Spike (S), nucleocapsid (N), and open reading frame 1 (ORF1).

### Viral RNA Library Preparation and Sequencing

We prepared amplicon libraries for viral genome sequencing using QIAseq FX DNA Library Kit and QIAseq SARS-CoV-2 Primer Panel (Qiagen, cat. no. 180475, cat. no. 333896) as instructed by the manufacturer’s manual, and the library was subsequently sequenced using Illumina platform. The adapter sequence used for each sample was compatible with Illumina sequencing instrument with 96-sample configurations (Qiaseq unique dual Y-adapter kit). The average insert length was in the 250–500 bp range. Prepared libraries were then pooled as a batch of 96 samples and sequenced using Illumina NextSeq 550 platform in 150 × 2 layout.

### Raw Data Preprocessing

Quality of the sequenced files was checked using FastQC tool (0.11.9) (Andrews, 2010), followed by removal of low quality bases (–nextseq-trim, Q < 20), Illumina Universal adapter sequence and reads with less than 30-bp length using Cutadapt (2.10) (Martin, 2011). To access the quantity of host genomic DNA and other contaminants, Kraken (2.0.9-beta) (Wood et al., 2019) was used, and the reports were summarized using Krona (2.7.1) (Ondov et al., 2011). All the files were then aligned to human genome (assembly version GRCh38) using HISAT2 (2.2.0) (Kim et al., 2015), and unmapped reads were extracted using SAMTOOLS (1.10) (Li et al., 2009) and converted to FASTQ format using BEDTOOLS (2.29.2) (Quinlan and Hall, 2010) bamToFastq option.

### Alignment With Viral Genome

The unmapped reads were then aligned to SARS-CoV-2 reference assembly (NCBI accession NC_045512) using HISAT2 (2.2.0) (Kim et al., 2015). Amplicon primes from the aligned file were removed using iVar (1.2.2) (Grubaugh et al., 2019) guided by Artic Network V3 primer scheme^1^. The aligned files were then deduplicated using Picard Tools (2.18.7)^2^. Alignment quality was checked using SAMTOOLS (1.10) (Li et al., 2009) flagstat option.

### Consensus Sequence Generation and Variant Calling

A consensus sequence for each isolate was generated using Bcftools (1.10) (Li, 2011) and SEQTK^3^. After generating a reference-based consensus sequence, we selected 202 genomes with less than 5% N’s and more than 10× coverage for phylogenetic and mutation analysis. Single-nucleotide variants were called and filtered (QUAL > 40 and DP > 20) using Bcftools (1.10) (Li, 2011). Effects of the filtered variants were annotated using SnpEff (4.5) (Cingolani et al., 2012). All of the consensus sequences were deposited in GISAID (Shu and McCauley, 2017); accession IDs are provided in Supplementary Table 2.

### Phylogenetic Analysis

Phylogenetic tree analyses of all the samples were performed using SARS-CoV-2 analysis protocol standards and tools provided by Nextstrain (Hadfield et al., 2018) pipeline. First, all the sequences are aligned against the WH01 reference genome using Augur wrapper of MAFFT (Katoh and Standley, 2013), and low-quality variant sites are masked from the alignment. The initial maximum likelihood tree was generated by the IQTREE2 tool (Nguyen et al., 2015) with 1,000 bootstraps. We have provided the maximum likelihood tree with bootstrap percentage marked for branches with ≥ 60 support and clade information in Supplementary Figure 3. Further refinement of the tree was done using the Augur refine command, and the tree was rooted using the reference sequence with timeline information incorporation using TimeTree (Kumar et al., 2017). To finalize the tree for Nextstrain auspice visualization the tree was annotated using ancestral traits, clades, nucleotide mutation and amino acid mutation. The resulting tree was visualized using an Auspice instance, and the visualization was refined using the ggtree R package.

We have used Nextstrain year-letter clade nomenclature that started with 19A and 19B branched by C8782T and T28144C nucleotide changes and was initially prevalent in Asia during initial outbreak. Later, 20A emerged in European outbreak from 19A parents having C3037T, C14408T, and A23403G as distinctive features. 20B emerged as a distinct clade in Europe with three consecutive mutations, e.g., G28881A, G28882A, and G28883C. Further the 20C emerged as a North America–specific clade with C1059T and G25563T nucleotide changes.

### Haplotype Network Analysis

For haplotype network analysis, we took a total of 287 (China 15, Germany 23, Italy 25, Saudi Arabia 23, Singapore 14, and South Korea) SARS-CoV-2 whole-genome sequences with less than 1% N and with collection date of March, April, and May from GISAID database. The selected samples are then aligned to the WH01 reference genome using MAFFT (Katoh and Standley, 2013). After filtering aligned sequences, we used POPART (Leigh and Bryant, 2015) software to generate haplotype network using a median joining method with 2,000 iterations.

### Modeling of the Protein Structures

The sequences of SARS-CoV-2 proteins (NPS3, NSP4b, NSP6, nucleocapsid, and Spike) were retrieved from NCBI. As most of the proteins do not have a three-dimensional (3D) structure in protein data bank (PDB), they were modeled using Modeler 9.21 (Webb and Sali, 2016). The suitable templates for modeling of the proteins (Supplementary Table 1) were selected by DELTA-BLAST (Boratyn et al., 2012) against the PDB proteins. One hundred models were generated for each of the proteins. The best model was selected based on the lowest DOPE score (Shen and Sali, 2006). The D614G mutant of the Spike protein was generated by Modeler 9.21. The loop in Spike protein (670–690) was refined using loop modeling procedure in Modeler by generating 100 loop models. The model with the lowest dope score was finally chosen for both the mutant and wild-type protein. Similarly, the host transmembrane serine protease 2 (TMPRSS2) was also modeled using Modeler. The PROCHECK (Laskowski et al., 1993) server was used to assess the stereochemistry of the generated models.

### Protein–Protein Docking

The standalone version of HADDOCK2.2 (van Zundert et al., 2016) was used to perform protein–protein docking with SARS-CoV-2 Spike protein with human TMPRSS2. The docking was conducted by the restraining of the receptor (Spike) and ligand (TMPRSS2) residues known to be at the interface Spike-TMPRSS2 interface. Specifically, residues within 5 Å of the reported cleavage site (Arg685, Ser686) (Hoffmann et al., 2020) of the Spike and catalytic triad of TMPRSS2 (H296, D345, and S441) and binding residue D435 were restrained to be at the docking interface. A total of 100 docking poses were generated and ranked based on the HADDOCK2.2 docking score (van Zundert et al., 2016), which is composed of van der Waals energy, electrostatic energy, restraints energy, etc. The best-ranked docking pose was visualized using Pymol.

### Statistical Analysis and Plotting

All the statistical analysis and plots were generated in R (3.6.1) statistical programming language using ggplot2, dplyr, reshape2, lubridate, ggsci, and ggpubr package available from CRAN and Bioconductor^4^ repository.

## Results

### Demographics, Clinical Status, and Travel History

The average age of the 225 patients was 30.98 ± 11.79 years with age range 1–75 years and median age of 30 years. The overall gender ratio of male-to-female was 201:24 with median age of male patients 31.75 and 24.5 years for female patients (Supplementary Figures 1A,B). Almost every female patient in this study reported no strong symptoms during sample collection, whereas in case of the male patients, we found that the numbers of symptomatic cases were almost the same with the number of asymptomatic cases (Supplementary Figure 1C). For the samples in our study, we did not observe any fatality in the patient group to our knowledge.

The majority of the patients (89%) disclosed their travel history during the sample collection procedure. We found that the majority of the patients included in the study were found to migrate from Gujarat, West Bengal, Tamil Nadu, Maharashtra, Delhi, Kerala, and Andhra Pradesh state in India (Supplementary Figure 1D). As most of the patients did not have any direct foreign travel (*n* = 1) history, the primary source of infection is local contacts in their workplaces, and this helped us to understand the COVID-19 strain diversity in the most affected states of India (Supplementary Figure 1D).

### Phylogenetic Analysis

**FIGURE 1:**
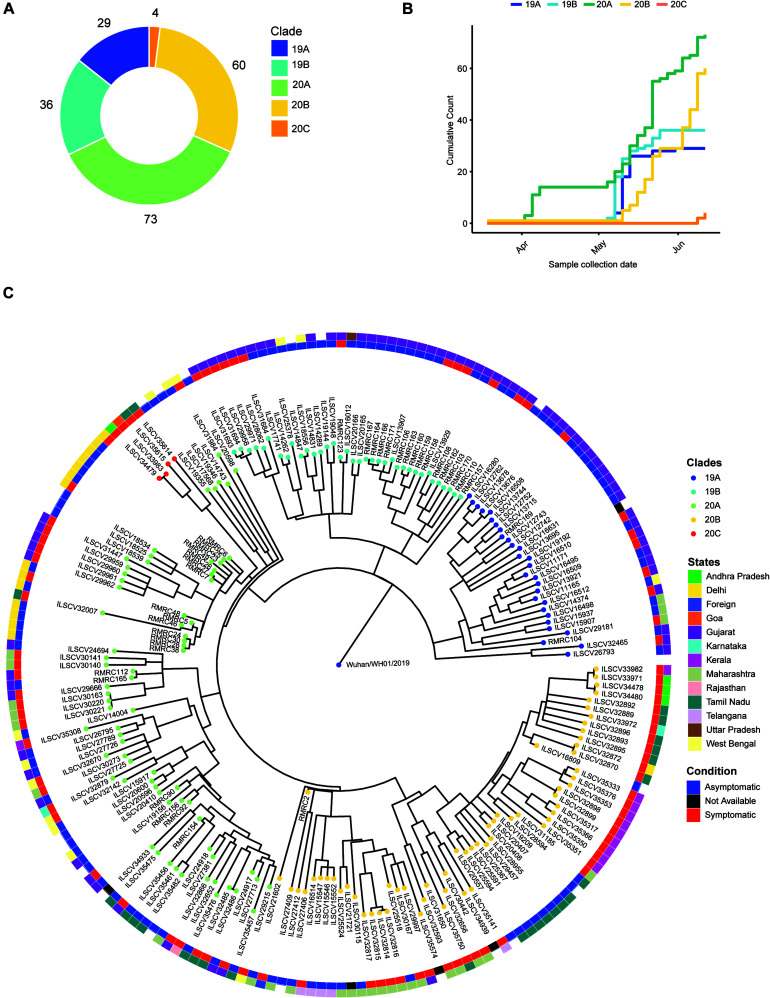
Phylogenetic analysis of the SARS-Cov genomes and their distribution into different Nextstrain defined new clades. **(A)** A donut chart representing the sequenced sample (*n* = 202) distribution across the clades (clade nomenclature obtained using Nextstrain). **(B)** Cumulative count of clades plotted against sample collection date showing abundance of clades with time. **(C)** Time tree (1,000 bootstraps) of the sequenced samples (*n* = 202) generated using Nextstrain time-tree pipeline overlaid with clinical status (condition, inner circle) of the patients during sample collection, place of migration (state, outer circle), and clade information (clades).

The phylogenetic analysis was carried out with 202 high-quality (<5% N’s) SARS-CoV-2 sequences that revealed presence of four major clades, i.e., 19A (*n* = 39), 19B (*n* = 36), 20A (*n* = 73), 20B (*n* = 60), and one minor clade 20C (*n* = 4) (Figure 1A). To understand the abundance of clades with time, we plotted cumulative counts of clades against the sample collection date and observed the introduction of clade 20A occurred in April 2020 with parallel emergence of 19A, 19B, and 20B in May (Figure 1B). After May, no new occurrence of clade 19A or 19B was observed in our data, but in mid-June, we started to observe emergence of 20C clade (Figure 1B). When we overlaid the clinical status information (asymptomatic vs. symptomatic), we observed that isolates from clade 20A, 20B, and 20C depicted a higher number of symptomatic patients (Figure 1C). Although without the information about predisposition of complication in patients, it is hard to establish an association between the mutations and the clinical manifestations.

To further understand the transmission of the virus in our patients, we performed phylogenetic analysis of our dataset with 1,042 high-coverage Indian SARS-CoV-2 whole-genome sequences (*N* < 1%) obtained from GISAID on July 12, 2020 (Supplementary Figure 1F). From the transmission map generated using Nextstrain, we observed that most of the sequences in 20A clades share sequence similarity with samples from Gujarat, which has very high prevalence of this clade (Supplementary Figure 1E). The 19A clade is very prevalent in Delhi and Telangana region and Tamil Nadu, but the 20B clade is only prevalent in the southern parts of India (Supplementary Figure 1E). Both 20A and 20B clades predominantly contain a mutation in protein coding sequence 23403A > G, and the mutation is traced back to the West European region in late January (Korber et al., 2020). The missense mutation in Spike protein coding gene causes a change in 614 D > G position of Spike protein and reported to increase the shedding of S1 subunit of the protein, which leads to the increased infectivity (Korber et al., 2020).

### Mutation Analysis

**FIGURE 2:**
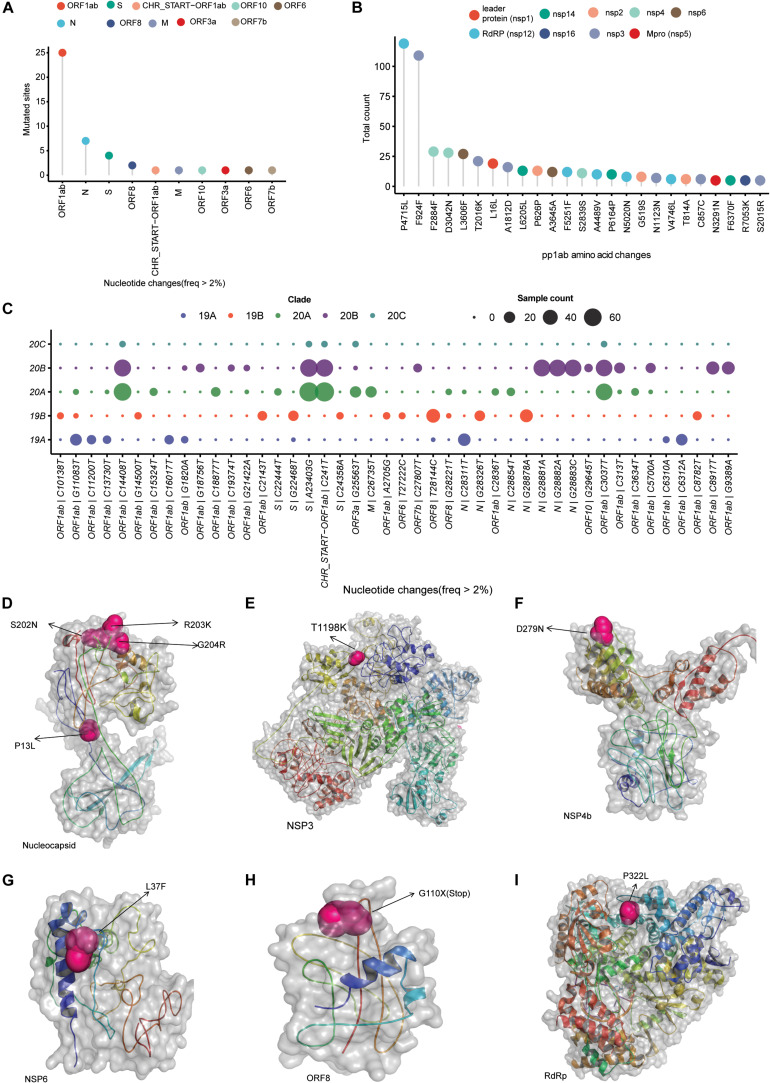
SARS-CoV-2 clade distribution and their prevalent mutation profiles. **(A)** Dot plot representing the number of single-nucleotide mutation (occurred in more than 2% of the samples) present in different genomic segments of SARS-CoV-2 genome. **(B)** The ORF1ab region codes for a polypeptide are later cleaved to several mature peptides. The dot plot represents the amino acid changes (location of amino acid acids as per location in polypeptide sequence) in the mature peptides of ORF1ab. **(C)** Clade-wise occurrence of nucleotide mutations with presence in more than 2% of sequenced samples (*n* = 202). Color of the dots represents the clade and size of the dots represents number of the samples showing presence of the single-nucleotide variant. **(D–I)** The mutation sites on the modeled structures of the SARS-CoV-2 proteins. The mutation site(s) of the NSP3, NSP4b, NSP6, RdRP, and nucleocapsid proteins are marked as sphere, while the rest of the structure is shown in cartoon representation.

After filtering out low-quality genomes with <5% N’s in the assembly and at least 10 × average coverage, we observed a total of 247 single-nucleotide variants from 202 SARS-CoV-2 isolates. Of these variants, 156 variants observed only in single isolates, 25 variants were classified as common variants with occurrence of more than 5% and 19 variants as rare with 2–5% occurrence (Supplementary Table 1). Among the common variants, the most frequent mutations are 23403 A > G (D614G, S gene), 241 C > T (5′ UTR), 14408 C > T (P4715L, RdRP gene), 3037 C > T (F942F, NSP3), 28881 G > A (R203K, N gene), 28882 G > A (R203R, N gene), 28883 G > C (G204R, N gene), and 28144 T > C (L84S, ORF8) with presence in more than 15% of all of our sequenced genomes (Supplementary Table 1). Plotting mutation diversity (>2%) in a clade-wise manner, we observed distinct mutation signatures in different clades. The isolates that were grouped in 19A clade depicted prevalence of mostly ORF1ab mutations with one distinct N gene C > T mutation at 28311 position (Figure 2C). In clade 19B samples, we observed a very distinct ORF8 T > C mutation at 28144 position, two N gene mutations at positions 28326 and 28878 with some ORF1ab mutation in lower frequency (Figure 2C). Clades 20A and 20B have almost similar mutation profile with major mutated positions 241 C > T mutation in leader sequence, ORF1ab 3037 C > T, ORF1ab 14408 C > T (RdRP), and S gene 23403 A > G mutations. The very distinct characteristic that we observed for 20B clade is three consecutive N gene mutations at positions 28,881, 28,882, and 28,883 (Figure 2C). Of these three mutations, two are missense mutations resulting in change of protein sequence. In clade 20A, we observed two mutations at ORF3a and M protein coding gene at position 25,563 G > T, 26,735 C > T but in less frequencies in comparison with other mutated sites (Figure 2C). Overall, to conclude, we summarized all the mutated sites present in all the samples and observed ORF1ab is the most mutated region, followed by N gene, S gene, and ORF8 in SARS-CoV-2 samples from India (Figure 2A). Among the ORF1ab mutations, 4,715 P > L change in nsp12, also known as RdRP (the viral RNA dependent RNA polymerase), was the most common one followed by a synonymous change (F924F) in nsp3 protein (Figure 2B).

To understand the impact of identified prominent missense mutations (present on > 10% of the samples) on the annotated viral proteins in our sequenced population, we used protein crystal structures and protein models as crystal structures were not available. The PROCHECK (Laskowski et al., 1993) results showed that the generated models have acceptable stereochemistry. First, we looked into the location of the mutated amino acid with respect to its functional domains as any change in the functional domain has high probability to perturb the protein function. The sites of mutation on the modeled protein structures (nucleocapsid, NPS3, NSP4b, NSP6, ORF8, and RdRP) are marked to indicate their location (Figures 2D–I).

### Haplotype Network Analysis

**FIGURE 3:**
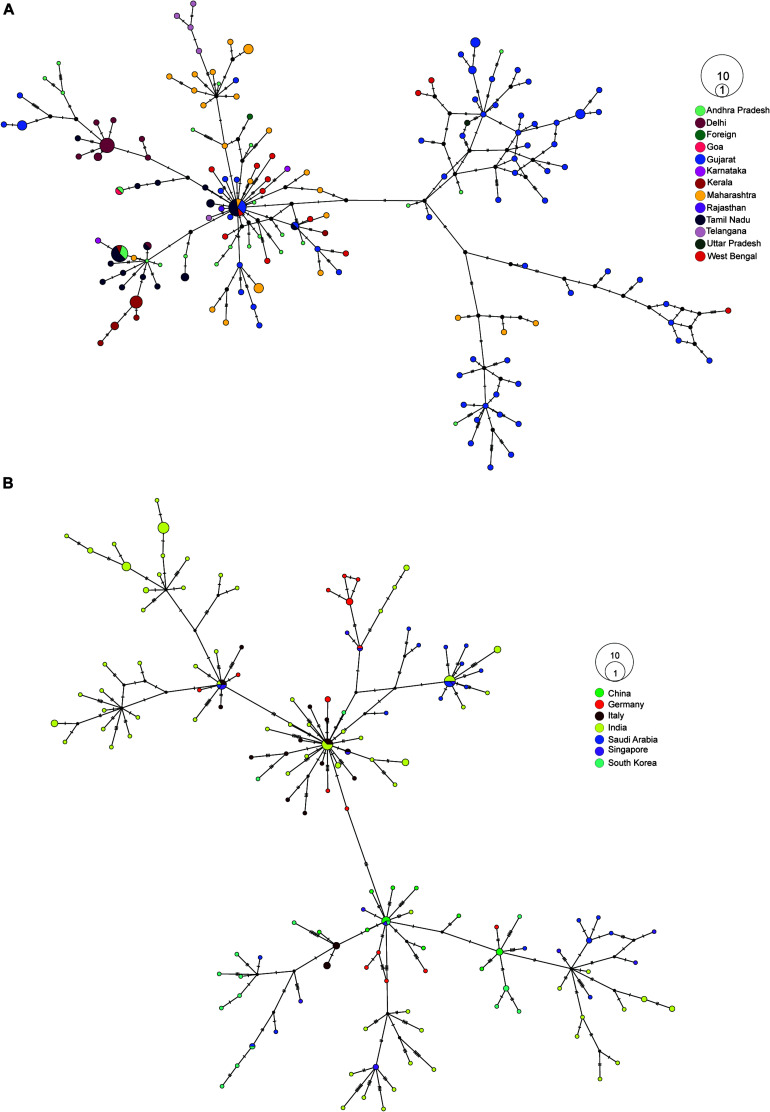
Haplotype network analysis of SARS-CoV-2 sequences. **(A)** Haplotype network of 202 SARS-CoV-2 whole-genome sequences from our dataset colored by their respective place of migration. **(B)** Haplotype network of 100 high-coverage SARS-CoV-2 genomes obtained from GISAID (China 15, Germany 23, Italy 25, Saudi Arabia 23, Singapore 14, South Korea 17) combined with 170 samples sequenced from Odisha with less than <5% N’s present in consensus sequence.

First, we created a median-joining haplotype network to look for transmission within India, and we observed two major branches (Supplementary Figure 2A). When we colored the sequences with clade information, the branches represented 19A and 20A, which later furcate into 19B, 20B, and 20C (Supplementary Figure 2A). When we overlaid migration information in the network, we observed the majority of 19A and 19B isolates migrated from Gujarat (Figure 3A). Migration of isolates identified in newly prevalent clades occurred from the southern path of India (Figure 3A). To understand the transmission source of SARS-CoV-2 infection in India, we constructed haplotype network using our sequencing data combined with genome sequences obtained from GISAID (China 15, Germany 23, Italy 25, Saudi Arabia 23, Singapore 14, and South Korea). From the haplotype network, we observed distinct clusters of genome sequences that were grouped in four major nodes. A large group of sequences clustered in two major haplotype clusters, one with genome sequences from China, Singapore, and South Korea and the other one with Italy, Saudi Arabia, and Germany with 2- to 4-nucleotide substitutions (Figure 3B). From the collection date, we observed that 20A clade, which is prevalent in Europe, became abundant in Odisha from April, representing the top cluster, whereas the bottom cluster represents samples belonging to clades 19A and 19B having a common origin in Southeast Asia (Figure 3B).

### Effect of D614G on Viral Load

**FIGURE 4:**
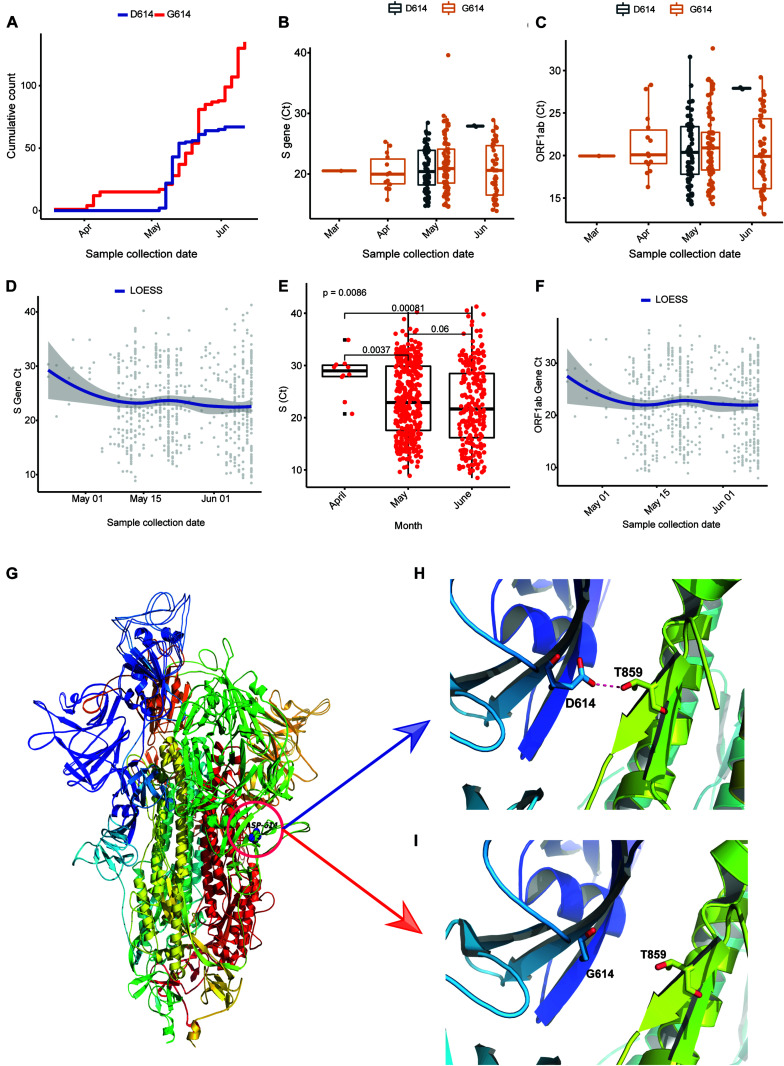
D614G in Spike gene increases infectivity portrayed by Ct values as a surrogate for viral load. **(A)** Cumulative count of the occurrence of D and G in 614 position of Spike protein in sequenced genomes (*n* = 202). **(B,C)** Ct value distribution of S gene and ORF1ab for the sequenced genomes (*n* = 202). **(D–F)** Ct value distribution of S gene and ORF1ab in all the positive samples tested at Institute of Life Sciences until June 17, 2020. **(G–I)** The superimposed 3D structures G614 mutant and wild-type Spike protein. **(G)** The mutant site is highlighted with a circle at 614 position. **(H)** The hydrogen bond (D614-T859) shown as dotted line between Spike S1 and S2 domain in wild type. **(I)** The hydrogen bond is lost as a result of D614G mutation.

To understand the prevalence and effect of G614 in our sequencing dataset, we plotted week-wise count (based on collection date) of D614 and G614 and observed that the occurrence of the mutation has been observed in early March (12th week), and the cumulative frequency increased over time (Figure 4A). We also assessed the frequency of D614G mutation from Covid19 Beacon database (CSIRO and CSIR-IGIB, access date: February 8, 2020) in global and all the SARS-CoV-2 sequence published from India, and the occurrence of G614 in Indian genomes is 76.31% where the global frequency is 43.8% (Supplementary Figure 2C). Every sample we sequenced as a part of the study was also checked for the levels of ORF1 and S gene using qPCR. The Ct obtained is also a direct indicator of viral load (lesser the Ct, higher the viral load) in the individual. When we plotted the Ct values of all sequenced samples, we observed that except week 21, the Ct values of the isolates having G614 mutation are less in comparison to isolates having D614 (Figures 4B,C). We also had Ct values (ORF1, S gene) of 637 positive isolates available as a partner institute of the COVID19 surveillance program in Odisha, India. Plotting the data against the date of sample collection for testing, we observed a sharp and significant (*p* < 0.05) decline (median ∼5 Ct change) in the Ct values (Figure 4E) from April 2020 to May 2020, and there was median ∼1 Ct change of S gene, as well as ORF1ab (Figures 4D–F) from the month of May to June 2020. In the case of ORF1 expression, we also observed a sharp change between April and May, but there is almost no change between May and June 2020 (Supplementary Figure 2A).

### Molecular Modeling Depicted Enhanced Interaction of D614G-Mutated Spike Protein With TMPRSS2 Protease

**FIGURE 5:**
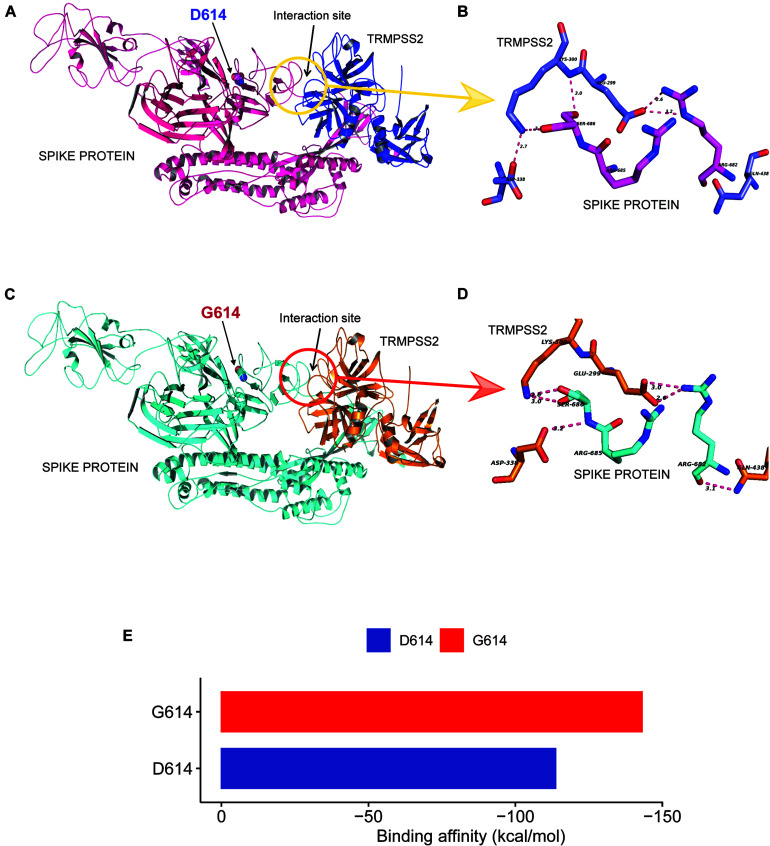
D614G change in Spike protein enhanced TMPRSS2 protease interaction that might be responsible for increased virus infectivity. **(A–D)** The docking study of TMPRSS2 with the wild-type (D614) and mutant (G614) Spike protein. The interaction site and the mutation position (614) is marked with an arrow. The hydrogen bond interactions are shown in pink dotted lines with distance marked in Å. **(A)** The overview of the docking site location on WT Spike protein, **(B)** the interactions between TMPRSS2 and wild type, **(C)** the overview of the docking site location on WT Spike protein, **(D)** the interactions between TMPRSS2 and mutant Spike protein, and **(E)** the average binding energy (kcal/mol) values for the top poses selected from five different clusters.

As reported in earlier publications, we also noticed D614G as a highly prevalent mutation in highly transmitted and evolved strains belonging to clades 20A and 20B in the Indian scenario; therefore, we carried out molecular modeling analysis. It was observed that the wild-type (D614) and mutated form (G614) of Spike protein showed a slightly different arrangement of structural elements near the mutation site, which is present in hinge region linking S1 and S2 domain, i.e., S1 furin cleavage site (Figure 4G). The S2 domain of the Spike protein is reported to interact with TMPRSS2 protease necessary for shedding of S1 domain for viral entry inside the host cells by facilitating the merging of virus with the host cell membrane. The proteolytic cleavage of Spike protein by TMPRSS2 results in the shedding of S1 domain, which is one of the key steps of the virus infection in host cells. Cryo–electron microscopy structures indicated that side chains of D614 protomer and T859 of the neighboring protomer form a hydrogen bond in between bringing together S1 domain with S2 (Lu et al., 2020; Walls et al., 2020). This substitution could modulate the glycosylation at N616 site as well, perturbing the interaction between the neighboring protomer. Our Spike protein model depicted this hydrogen bonding between D614-T859 of S1 and S2 domain (Figure 4H). The mutation of D614 to G614 eliminates this side-chain hydrogen bonding between S1 and S2 domain (Figure 4I), leading to increased main-chain flexibility enabling a more favorable orientation of Q613, possibly facilitating cleavage by TMPRSS2 by perturbing its affinity with the S1-furin cleavage site. It has also been proposed that D614 forms an intrasalt bridge with R646, which makes the conformation unfavorable for S1 association with S2 domain (Zhang L. et al., 2020). Interestingly, the protein docking analysis depicted better hydrogen bonding interactions between the Spike protein cleavage sites (Arg685, Ser686) with the catalytic triad of TMPRSS2 in mutant condition as compared to wild-type. In the case of mutant Spike protein, the Arg682 and residues at primary cleavage site of Spike protein (Arg685 and Ser686) formed six hydrogen bonding interactions with Glu299, Lys300, Asp338, and Gln438 residues of TMPRSS2 (Figures 5A,B), whereas in the D614 wild-type form there were five hydrogen bonds observed between the cleavage site of Spike protein S2 domain and TMPRSS2 (Figures 5C,D). The binding energy was observed to be better for the G614 mutant (-143.03 kcal/mol) as compared to that of the wild type (−113.67 kcal/mol), indicating better binding of TMPRSS2 with the mutated Spike protein (Figure 5E).

## Discussion

The pattern of COVID-19 pandemic spread in India is quite different from all over the world in terms of slow transmission and lower mortality rates in the beginning. Therefore, it was extremely important to understand the transmission dynamics of SARS-CoV-2 and its evolution during different phases of disease in India. The mutations that accumulate in the virus genome with time act as a molecular clock that can provide insight into emergence and evolution of the virus. These analyses could be helpful to prevent or control the transmission of the virus. To track the COVID-19 outbreak and understand the genomic clades of SARS-CoV-2 prevalent in India as compared to rest of the world, we performed the genome sequencing of oropharyngeal and nasopharyngeal swabs samples collected from individuals migrated from different regions of India to the state of Odisha after the reported incidences of COVID-19 pandemic in India.

For SARS-CoV-2 genome sequencing, we did amplicon-based sequencing for 225 SARS-CoV-2 genomes to capture the major clade diversity in India, of which we selected 202 SARS-CoV-2 sequences based on their assembly coverage (<5% gap) for further detailed analysis. We selected migrant groups from different parts of the country so that diversity of COVID-19 prevalent in the country could be captured in the phylogenetic analysis to understand disease transmission and virus evolution with time. The sequenced samples represent migratory populations from North, East, West, and Southern parts of India. Our analysis depicted that all the genomes analyzed in our study were grouped into four major clades, 19A, 19B, 20A, and 20B, according to the new Nextstrain clade nomenclature. Clade 19A is the Wuhan clade from China. Interestingly, we were able to capture occurrence of a rare clade with very less occurrence in India, i.e., 19B with 17% (*n* = 36) in our samples. This clade was found to be prevalent in East and Southeast Asia during the early outbreak of the pathogen. This confirmed that the foundation of the source of early outbreak infection in India also came from Southeast Asian countries. The migration information for these early collected samples were not well defined; therefore, it was difficult for us to pinpoint exactly which Southeast Asian country the transmission of 19B started. The mutation analysis depicted that both 19A and 19B clades almost evolved in parallel as prevalent mutations in both the clades are highly variable. On the other side, the phylogenetic analysis showed that clades 20A and 20B evolved quite rapidly in the Indian population and are a major source of disease transmission in the country, whereas the 20C strain is rarely detected and appeared to be less adapted or somehow contracted by contact tracing at early stages of infection. The haplotype network construction also pointed to the later strains belonging to 20A; 20B clades originated from Western Europe and transmitted directly or via Saudi Arabia are mostly prevalent in the southern and western part of India. In the clade, we also observed a very less frequent clade 20C, prevalent only in a handful of the Middle Eastern countries making up a marginal portion (*n* = 4) of our sequenced samples. This suggests the requirement of constant monitoring of SARS-CoV-2 with sequencing technology to understand the source of infection and design prevention mechanisms such as strategic lookdowns and region specific travel restrictions.

The fitness of the virus strain and its transmission depend on the adaptive mutations that it acquires with time. From initial observation, we have seen a 10.34% occurrence of C6312A, which has been associated with an India-specific clade called I/A3i (Banu et al., 2020). The mutation is dominant only India as the global frequency of the mutation is around 1.2% according to the Covid19 Beacon database. We found that four common variants, i.e., 241 C > T in the UTR region, 3,037 C > T in NSP3 gene, 14,408 C > T in the NSP12, and 23,403 A > G in S gene coevolved mostly in the 20A and 20B clades. As 20A and 20B clade frequency increased with time in the population, which indicates that these strains have some selective advantage with time for increased transmission. It has been reported that the leader sequence present in the UTR region of positive strand RNA viruses like SARS-CoV is important for the replication and strand switching to generate negative strands (Kim et al., 2020). This mutation in leader sequence is 20 nucleotides upstream of translation start site of ORF1ab gene. Therefore, it might be providing an advantage to virus in preventing stem-loop generation required during strand switching by RdRP, which needs further experimental evaluation. At the same time, several reports documented that 23,403 A > G (D614G) missense mutation in the Spike protein enhanced the infectivity rate of the virus. One of the reports showed that the shedding of S1 domain of Spike protein changes due to change in the hydrogen bonding between S1 and S2 domains. We observed in our protein modeling analysis that TMPRSS2 binding to Spike protein is enhanced by this mutation of aspartic acid to glycine (D614G) as it resulted in increased hydrogen bonding interactions. This change enhances the interaction of TMPRSS2 with the S2 domain, which is important for the cleavage of the S1 domain and virus entry into cells by facilitating its entry into the host cells. The overall primary *in silico* docking study showed that mutant Spike protein has a greater number of hydrogen bonds with TMPRSS2 at the cleavage site as compared to the wild type, resulting in better docking energy. Overall analysis indicates that the breakage of a hydrogen bond as a result of the mutation may facilitate greater cleavage of the mutant Spike protein as compared to the wild type. All the sequenced genomes were submitted in the GISAID database.

## Odisha COVID-19 study group (Author names are arranged in alphabetical manner)

Arvind Kumar Singh, Baijayantimala Mishra, Banajini Parida, Binod Kumar Patro, D. P. Dogra, Dasarathi Das, Deepa Prasad, Dhaneswari Jena, Dharitri Mohapatra, Dinesh Prasad Sahu, Durga Madhab Satapathy, Durgesh Prasad Sahoo, Jayanta Panda, Jaya Singh Khatri, Kaushik Mishra, Manoranjan Satpathy, Nirupama Chaini, Roma Rattan, Sadhu Panda, Sangeeta Das, Somen Kumar Pradhan, Srikanta Kanungo, Sriprasad Mohanty, Subrata Kumar Palo.

## ILS COVID-19 group (Author names are arranged in alphabetical manner)

Aditi Chatterjee, Adyasha Mishra, Ajit Kumar Singh, Amrita Ray, Ankita Datey, Aliva Minz, Ashish Yadav, Auromira Khuntia, Anshuman Dixit, Debyashreeta Barik, Deepak Singh, Eshna Laha, Hiren G. Dodia, Jeky Chawla, Kautilya Jena, Kaushik Sen, Niyati Das, Omprakash Shriwas, P. M. Vaishali, Parej Nath, Paritosh Nath, Prabhudutta Mamidi, Priyanka Mohapatra, Rahul Das, Rina Yadav, Sachikanta Rout, Saikat De, Sanchari Chatterjee, Sandhya Suranjika, Satyaranjan Sahoo, Shamima Ansari, Shifu Aggarwal, Shiva Pradhan, Sivaram Krishna, Sneha Dutta, Soumendu Mahapatra, Soumyajit Gosh, Subhabrata Barik, Sudhir Boral, Supriya Suman Keshry, Swatismita Priyadarshini, Tsheten Sherpa.

## Data Availability Statement

In the data availability statement the name of the Online repository is GISAID (https://www.gisaid.org/) accession ids are available in Supplementary Table 2.

## Ethics Statement

The studies involving human participants were reviewed and approved by the Institutional Human Ethics Committee, Institute of Life Sciences. Written informed consent to participate in this study was provided by the participants’ legal guardian/next of kin.

## Author Contributions

AP, SP, SR, and JT planned and designed the study. AJ, SM, MP, VB, PS, BS, NS, DS, AD, SK, KA, SSm, and JS did the experiments. SC, GS, RD, SSe, RS, TB, and PP coordinated sampling and COVID-19 testing analysis. AG and SR did the genomic data analysis. SK and AD performed protein modeling and docking analysis. All authors have read and approved the manuscript.

## Conflict of Interest

The authors declare that the research was conducted in the absence of any commercial or financial relationships that could be construed as a potential conflict of interest.

## Footnotes

1 https://github.com/artic-network/artic-ncov2019/tree/master/primer_schemes/nCoV-2019/V3

2 https://broadinstitute.github.io/picard/

3 https://github.com/lh3/seqtk

4 https://CRAN.R-project.org/package=tidyvers

## Supplementary Material

Supplementary Figure 1 Patient demographics, transmission map and diversity of COVID-19 in India. **(A–D)** Represents the distribution of gender, age, clinical status and geographical location of sampled patients (*n* = 225). **(E–F)** Transmission map and Timetree of the 1,042 high coverage Indian SARS-CoV-2 whole genome sequences (*N* < 1%) obtained from GISAID on 12th July, 2020.

Supplementary Figure 2 ORF1ab Ct values in tested positive samples (*n* = 637) and haplotype network of sequenced data colored by clade. **(A)** ORF1ab Ct values in tested positive samples (*n* = 637) binned in their respective collection months. **(B)** Haplotype network of 202 SARS-CoV-2 whole genome sequences from our dataset colored by their respective clade. **(C)** Comparison of D614G mutation in global and Indian SARS-CoV-2 sequences.

Supplementary Figure 3 Maximum likelihood tree of SARS-CoV-2 genomes (*n* = 202). **(A)** Maximum likelihood tree of SARS-CoV-2 genomes with 1,000 bootstraps and branch support values with =60 threshold.

Supplementary Table 1 Information about patients, List of single nucleotide mutations and the annotated genes, corresponding amino acid changes and its predicted impact on protein function.

Supplementary Table 2 Accession ids of GISAID submissions.
