# Supplementary figures and images for: Analysis of Indian SARS-CoV-2 Genomes Reveals Prevalence of D614G Mutation in Spike Protein Predicting an Increase in Interaction With TMPRSS2 and Virus Infectivity
Source: Front Microbiol. 2020 Nov 23;11:594928. doi: 10.3389/fmicb.2020.594928 (PMC7732478; doi:10.3389/fmicb.2020.594928)

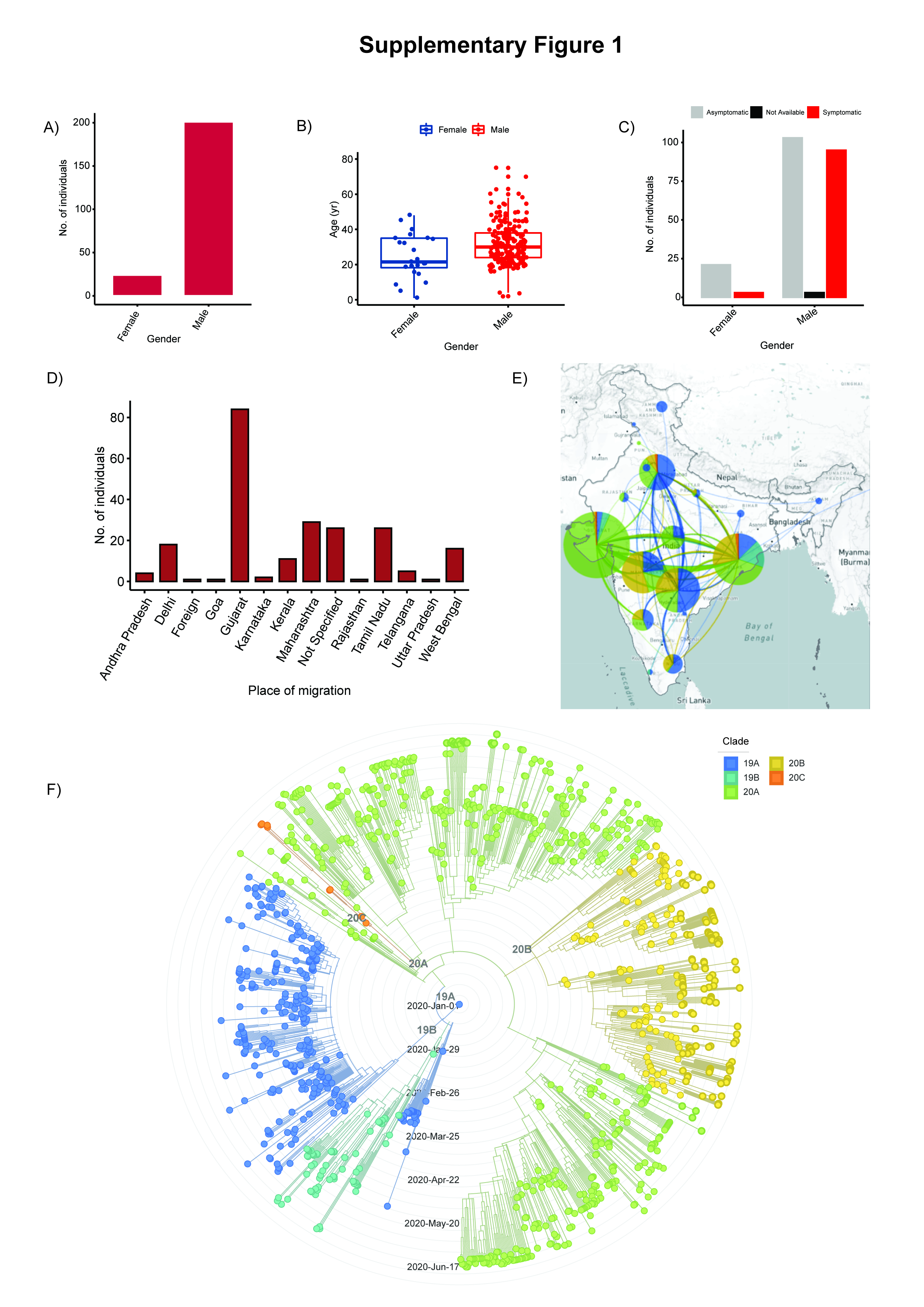

Supplement: Supplementary Figure 1 — Patient demographics, transmission map and diversity of COVID-19 in India. (A–D) Represents the distribution of gender, age, clinical status and geographical location of sampled patients (n = 225). (E–F) Transmission map and Timetree of the 1,042 high coverage Indian SARS-CoV-2 whole genome sequences (N < 1%) obtained from GISAID on 12th July, 2020. [file Image_1.TIF]

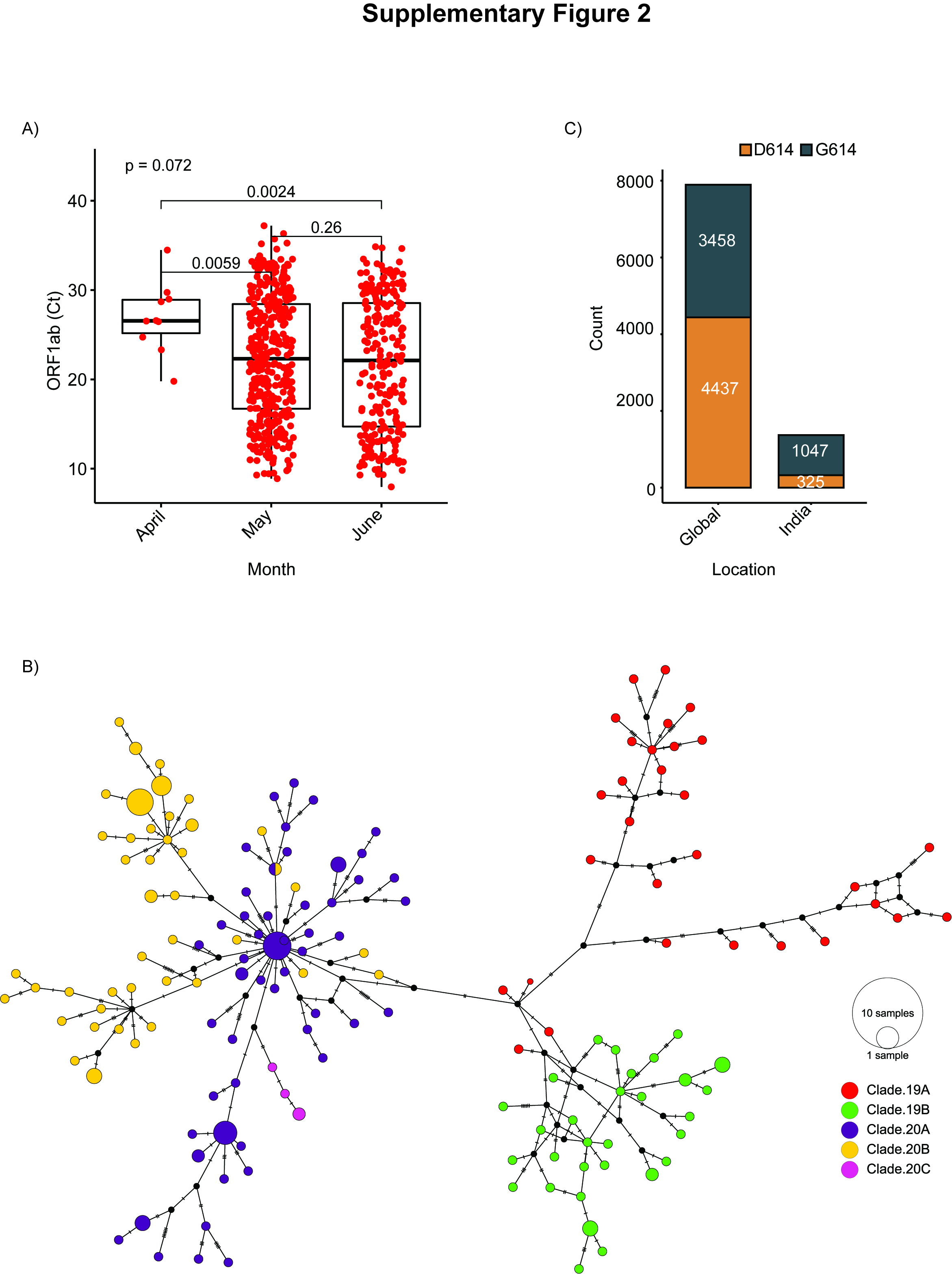

Supplement: Supplementary Figure 2 — ORF1ab Ct values in tested positive samples (n = 637) and haplotype network of sequenced data colored by clade. (A) ORF1ab Ct values in tested positive samples (n = 637) binned in their respective collection months. (B) Haplotype network of 202 SARS-CoV-2 whole genome sequences from our dataset colored by their respective clade. (C) Comparison of D614G mutation in global and Indian SARS-CoV-2 sequences. [file Image_2.TIF]

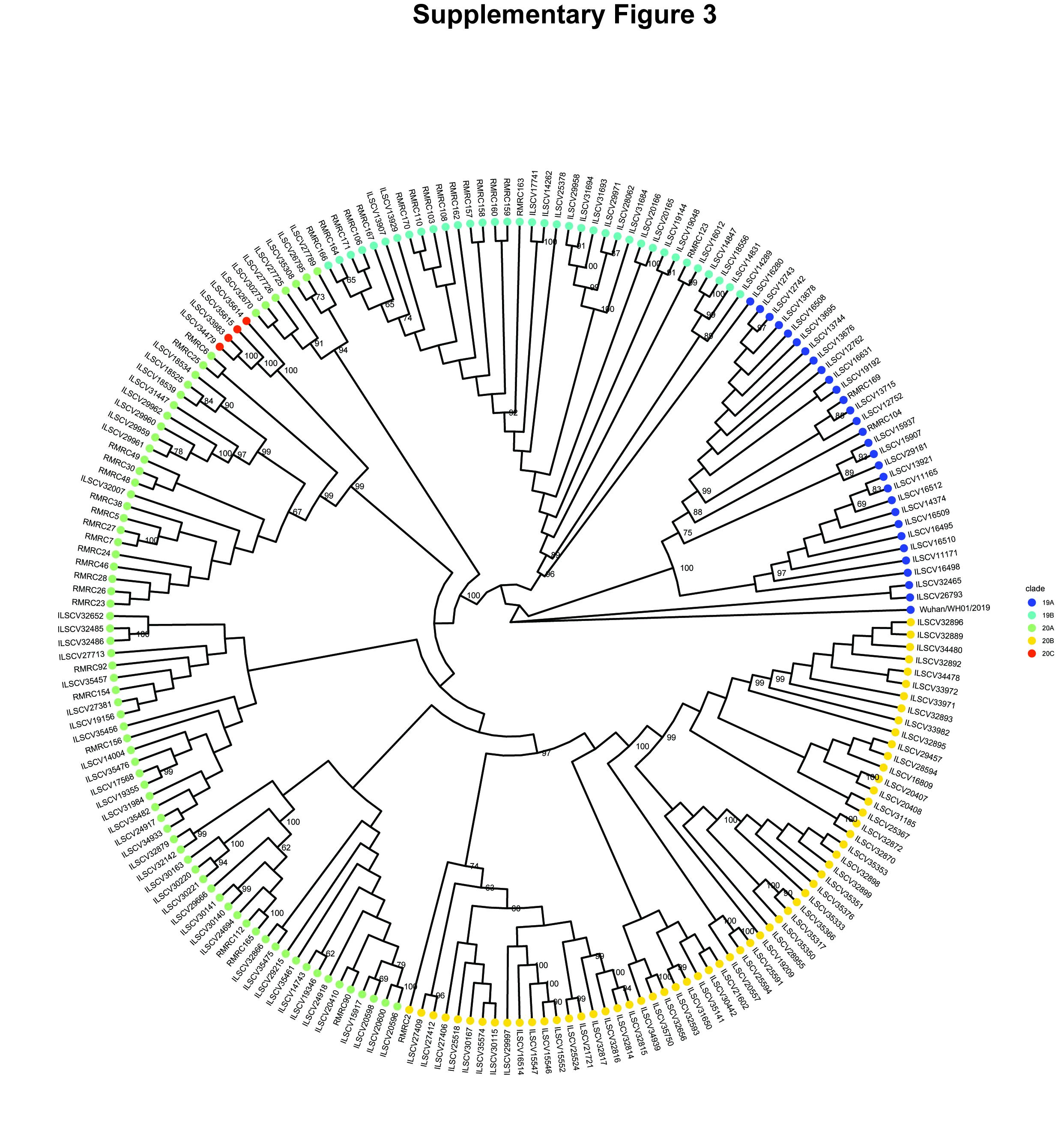

Supplement: Supplementary Figure 3 — Maximum likelihood tree of SARS-CoV-2 genomes (n = 202). (A) Maximum likelihood tree of SARS-CoV-2 genomes with 1,000 bootstraps and branch support values with =60 threshold. [file Image_3.TIF]
